# Influenza vaccination trend and related factors among patients with diabetes in Korea: Analysis using a nationwide database

**DOI:** 10.3389/fendo.2023.1077846

**Published:** 2023-02-03

**Authors:** Dong-Hwa Lee, Bumhee Yang, Seonhye Gu, Eung-Gook Kim, Youlim Kim, Hyung Koo Kang, Yeong Hun Choe, Hyun Jeong Jeon, Seungyong Park, Hyun Lee

**Affiliations:** ^1^ Division of Endocrinology and Metabolism, Department of Internal Medicine, Chungbuk National University College of Medicine and Chungbuk National University Hospital, Cheongju, Republic of Korea; ^2^ Division of Pulmonary and Critical Care Medicine, Department of Internal Medicine, Chungbuk National University Hospital, Chungbuk National University College of Medicine, Cheongju, Republic of Korea; ^3^ Department of Epidemiology and Health Informatics, Korea University, Seoul, Republic of Korea; ^4^ Department of Biochemistry, Chungbuk National University College of Medicine, Cheongju, Republic of Korea; ^5^ Division of Pulmonary and Allergy, Department of Internal Medicine, Konkuk University Medical Center, Konkuk University School of Medicine, Seoul, Republic of Korea; ^6^ Division of Pulmonary and Critical Care Medicine, Department of Internal Medicine, Inje University Ilsan Paik Hospital, Inje University College of Medicine, Goyang, Republic of Korea; ^7^ Research Institute of Clinical Medicine of Chonbuk National University-Biomedical Research Institute of Chonbuk National University Hospital, Jeonju, Republic of Korea; ^8^ Department of Internal Medicine, Chonbuk National University Hospital-Chonbuk National University Medical School, Jeonju, Republic of Korea; ^9^ Division of Pulmonary Medicine and Allergy, Department of Internal Medicine, Hanyang University College of Medicine, Seoul, Republic of Korea

**Keywords:** diabetes mellitus, influenza, vaccination, prevalence, epidemiology

## Abstract

**Background:**

Subjects with diabetes are at higher risk of serious influenza-related complications. We aimed to investigate the yearly trend of influenza vaccination and factors associated with being unvaccinated for influenza in subjects with diabetes using a nationwide observational study performed within the recent decade.

**Methods:**

Among 105,732 subjects from the Korea National Health and Nutrition Examination Survey between 2007 and 2019, 8,632 with diabetes were included. We investigated the yearly trend of influenza vaccination and factors associated with being unvaccinated for influenza.

**Results:**

During the study period, the prevalence of influenza vaccination in subjects with diabetes showed a tendency to increase every year, reaching almost 60% in 2019, which was higher than the rate in subjects without diabetes. Younger age (adjusted hazard ratio (aHR) [95% CI] 11.29 [8.63–14.75] for < 50 years; 6.16 [5.21–7.29] for 50–65 years), male (aHR 1.67 [1.52–1.87]), current smoker (aHR 1.31 [1.00–1.72], lower-income status (aHR 1.46 [1.17, 1.84]), and high education level (aHR 1.30 [1.01–1.67]) were associated with being unvaccinated. Also, a poorer glycemic control with HbA1c ≥ 9% was found to be correlated with unvaccinated status (aHR 1.48 [1.15–1.90]).

**Conclusion:**

The influenza vaccination rate is still unsatisfactory in subjects with diabetes. Young age, males, low-income level, high education level, and poor glycemic control were associated with unvaccinated status. Considering the risk-benefits of influenza vaccination in patients with diabetes, physicians should make an effort to increase vaccination rates, especially in low vaccination rate groups.

## 1 Introduction

Diabetes is a common metabolic disorder affecting the entire body (1). It is one of the top 10 causes leading to mortality and is responsible for over 55.4 million deaths/year worldwide (2). Diabetes increases the susceptibility of infections and worsens their treatment outcomes (3, 4). Of various infections, respiratory infections have been shown to impose a substantially increased risk of morbidity and mortality on subjects with diabetes (5, 6). Thus, preventing respiratory infection in subjects with diabetes would be important to improve the treatment outcomes of diabetes.

Influenza is one of the commonly identified respiratory viruses (7). Influenza can cause serious illness in people with certain underlying chronic conditions including diabetes (8, 9). For example, diabetes triples the risk of influenza-related hospitalization, quadruples the risk of admission to an intensive care unit, and doubles the risk of fatal outcomes (10, 11). Accordingly, subjects with diabetes are strongly recommended to be vaccinated for influenza (12, 13). The significant benefits of influenza vaccination have been well demonstrated in its efficacy to reduce complications, hospitalizations, and death in subjects with diabetes (11, 14).

Information about coverage of influenza vaccination and factors affecting vaccination would be helpful to make health policy to enhance influenza vaccination in subjects with diabetes. However, despite the importance of influenza vaccination in subjects with diabetes (11, 15), few studies addressed this important issue. Hence, we aimed to investigate the yearly trend of influenza vaccination and factors associated with being unvaccinated for influenza in subjects with diabetes using a nationwide observational study performed within the recent decade.

## 2 Methods

### 2.1 Study population

**Figure 1:**
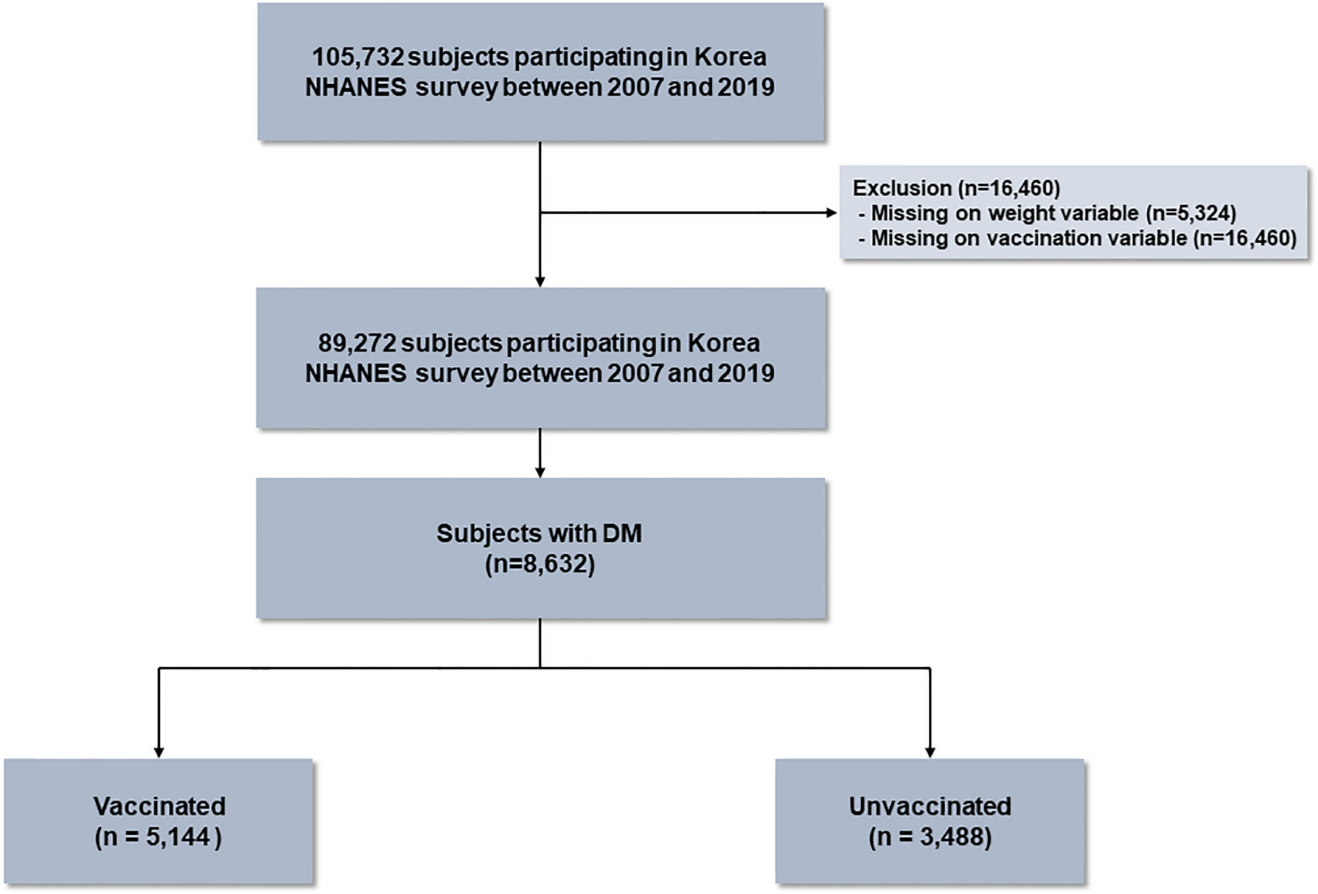
Flow chart of the study population.

The Korea National Health and Nutritional Survey (NHANES) is a population-based nationwide survey to assess the health and nutritional status of the noninstitutionalized population of Korea, and conducted by Korea Disease Control and Prevention (KCDC). We used data from the Korea NHANES IV (2007–2009), V (2010–2012), VI (2014–2015), VII (2016-2018), and VIII (2019). The data from Korea NHANES V (2013) was excluded because the vaccination data was not available. The study population was investigated based on a stratified multi-stage sampling method. During the 13 years, there were 105,732 participants. Of these, participants with missing on survey weight variable (n = 5,324) or vaccination variable (n = 16,460) were excluded, and 89,272 subjects were included in this study. Of the eligible participants, 8,632 (9.7%) subjects had diabetes, who were classified into two groups based on influenza vaccination status: vaccinated (n = 5,144) and unvaccinated (n = 3,488) (**Figure 1**). The study protocol was approved by the Institutional Review Board of Chungbuk National University Hospital (application no.2022-03-003).

### 2.2 Exposure: Diabetes

The main exposure of this study was diabetes. Diabetes was defined as any presence of the following criteria: 1) history of physician-diagnosis of diabetes. 2) fasting glucose level ≥ 126 mg/dL, 3) hemoglobin A1c (HbA1c) ≥ 6.5% (16), or 4) participants who reported taking oral hypoglycemic agents or administering insulin were classified as having diabetes (17).

### 2.3 Outcomes: Influenza vaccination

The main outcomes of our study were to evaluate the trend of influenza vaccination and to explore factors associated with influenza vaccination in patients with diabetes. Influenza vaccination was investigated using the following question: “Have you ever been vaccinated against influenza in the past year?”

### 2.4 Measurements

Available data on age, sex, body mass index (BMI), smoking history, marital status, income, education, respiratory symptom physical activity, and the EuroQoL five dimensions questionnaire (EQ-5D) index values were obtained from the Korea NHANES database. Regular walking was defined as a person who walked at least five times a week for more than 30 minutes at a time. The EQ-5D index values, used to measure the quality of life, range between 0 (worst imaginable health state) and 1 (best imaginable health state). Comorbidities of pulmonary tuberculosis, cardiovascular disease including myocardial infarction or angina, stroke, and chronic kidney disease were self-reported based on previous physician diagnosis (18, 19). Hypertension was defined as a self-reported physician diagnosis, the use of antihypertensive medication, a systolic blood pressure ≥ 140 mmHg, or a diastolic blood pressure ≥ 90 mmHg (20, 21). Dyslipidemia was defined as a self-reported physician diagnosis, the use of lipid-lowering medication, total cholesterol ≥ 240 mg/dL, or fasting triglyceride ≥ 200 mg/dL (19, 21). The data of HbA1c was obtained from a general health examination.

### 2.5 Statistical analysis

All analysis was performed using survey commands in STATA 15.1 version (StataCorp LP, College Station, TX, USA) to account for the complex sampling design and survey weights. All data are presented as a weighted percentage with standard errors (SE). The data were compared using Student’s t-test for continuous and Pearson’s χ2 test for categorical variables. To evaluate factors associated with influenza vaccination status in patients with diabetes, both univariable and multivariable logistic regression analyses were performed. Multivariable analysis was adjusted for age, sex, BMI, smoking status, marital status, income, regular walking, respiratory symptoms, physical limitation, EQ-5D, HbA1c and comorbidities (pulmonary tuberculosis, hypertension, dyslipidemia, chronic kidney disease, cardiovascular disease, and stroke). All tests were two-sided, and p-values < 0.05 were considered statistically significant differences.

## 3 Results

### 3.1 Baseline characteristics

The baseline characteristics of the study population are presented in **Table 1**. Of 8,632 participants, about 60% of participants were vaccinated (vaccinated group) and 40% were unvaccinated (unvaccinated group). Compared with vaccinated group, unvaccinated group was younger (mean 53.2 years vs. 65.2 years), had a lower proportion of female (36.7 vs. 53.5%), and a higher proportion of overweight or obesity (52.9 vs. 49.4%) and current smoker (31.5 vs. 16.3%) (P < 0.001 for all variables). Fasting plasma glucose and HbA1c levels were significantly higher in unvaccinated group compared with vaccinated group (148.9 vs. 137.3 mg/dL and 7.4 vs. 7.1%, respectively) (P < 0.001 for both). Regarding comorbidities, vaccinated group had a higher proportion of hypertension (67.6 vs. 51.0%), cardiovascular disease (8.3 vs. 3.9%), stroke (6.9 vs. 3.5%), and chronic kidney disease (15.6 vs. 5.8%) compared with unvaccinated group (P < 0.001 for all). The proportion of individuals with dyslipidemia was similar in both groups (77.0 vs. 78.0%, P = 0.392).

**Table 1:** Baseline characteristics of the study population in patients with diabetes mellitus.

	Total (N = 8,632)	Vaccinated (N = 5,144)	Unvaccinated (N = 3,488)	P value
Age, years	59.4 (0.20)	65.2 (0.24)	53.2 (0.25)	<0.001
Sex, female	45.5 (0.62)	53.5 (0.83)	36.7 (0.92)	<0.001
BMI, kg/m^2^				0.016
Underweight (< 18.5)	1.4 (0.15)	1.3 (0.18)	1.5 (0.24)	
Normal weight (18.5–24.9)	47.5 (0.64)	49.3 (0.83)	45.6 (0.98)	
Overweight/obesity (≥ 25)	51.1 (0.65)	49.4 (0.83)	52.9 (0.99)	
Smoking status				<0.001
Non-smoker	49.1 (0.65)	56.2 (0.84)	41.4 (0.98)	
Past smoker	27.3 (0.59)	27.5 (0.73)	27.2 (0.92)	
Current smoker	23.6 (0.59)	16.3 (0.66)	31.5 (0.96)	
Marital status				<0.001
Unmarried	5.6 (0.37)	2.5 (0.32)	8.9 (0.69)	
Married	73.5 (0.63)	69.4 (0.82)	78.0 (0.89)	
Widowed/Separated/Divorced	20.9 (0.54)	28.1 (0.80)	13.1 (0.63)	
Income				<0.001
Low	30.0 (0.66)	27.4 (0.79)	32.8 (1.01)	
Intermediate	47.7 (0.71)	48.3 (0.90)	47.1 (1.06)	
High	22.3 (0.61)	24.4 (0.82)	20.0 (0.83)	
Education				<0.001
Elementary school graduate	37.3 (0.65)	48.8 (0.88)	24.9 (0.85)	
Middle/High school graduate	44.2 (0.67)	37.9 (0.85)	51.0 (1.04)	
College graduate	18.5 (0.58)	13.3 (0.64)	24.2 (0.94)	
Regular walking	78.7 (0.55)	77.3 (0.71)	80.1 (0.81)	0.010
Symptoms				
Any respiratory symptoms	8.5 (0.45)	9.1 (0.59)	7.9 (0.67)	0.180
Cough	4.0 (0.32)	4.5 (0.45)	3.3 (0.45)	0.051
Sputum	7.5 (0.43)	7.8 (0.56)	7.1 (0.63)	0.356
Physical limitation	17.4 (0.50)	21.7 (0.71)	12.7 (0.64)	<0.001
Quality of life				
EQ-5D	0.9 (0.00)	0.9 (0.00)	0.9 (0.00)	<0.001
HbA1c	7.2 (0.02)	7.1 (0.02)	7.4 (0.03)	<0.001
Glucose levels, Fasting	142.9 (0.60)	137.3 (0.70)	148.9 (1.01)	<0.001
Comorbidities				
Pulmonary TB	5.3 (0.29)	5.9 (0.38)	4.7 (0.45)	0.037
Hypertension	59.6 (0.67)	67.6 (0.81)	51.0 (1.01)	<0.001
Dyslipidemia	77.5 (0.55)	77.0 (0.72)	78.0 (0.83)	0.392
Cardiovascular disease^*^	6.2 (0.28)	8.3 (0.43)	3.9 (0.36)	<0.001
Stroke	5.3 (0.28)	6.9 (0.41)	3.5 (0.36)	<0.001
Chronic kidney disease	10.9 (0.39)	15.6 (0.60)	5.8 (0.44)	<0.001

Data are presented as a weighted percentage with standard errors.

^*^Cardiovascular disease was defined as myocardial infarction or angina.

BMI, body mass index; EQ-5D, EuroQol-5-Dimension; TB, tuberculosis.

### 3.2 Influenza vaccination trend

**Figure 2:**
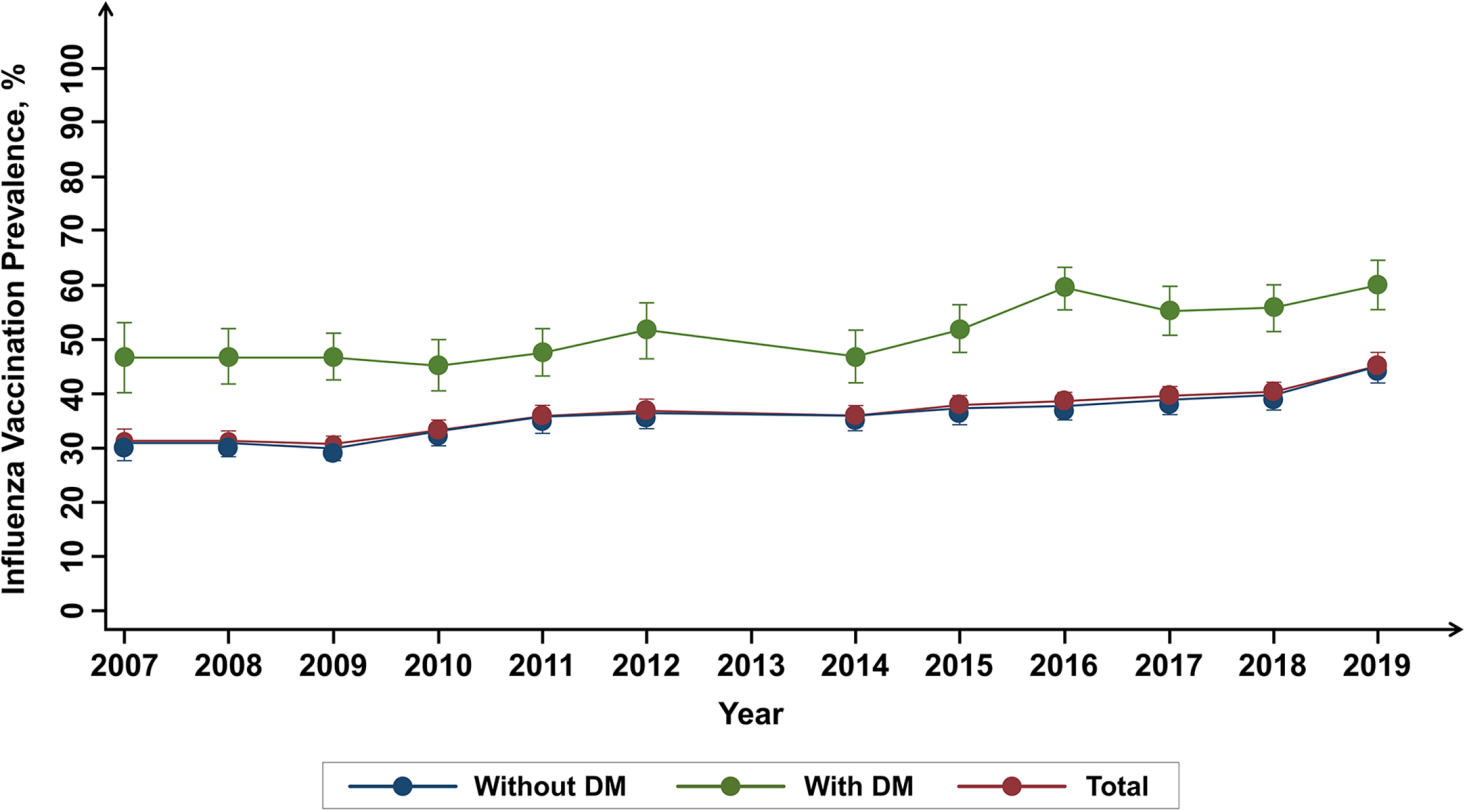
Influenza vaccination trends over 13 years according to the diabetes mellitus history.

The 13-year trend of influenza vaccination according to diabetes is summarized in **Figure 2**. Regardless of diabetes, increasing tendency was observed (from 30.9% in 2007 to 45.8% in 2019 for total population; from 46.54% in 2007 to 59.9% in 2019 for participant with diabetes; from 29.8% in 2007 to 44.1% in 2019 for participants without diabetes). Individuals with diabetes showed a significantly higher prevalence of influenza vaccination compared to those without diabetes (P < 0.001).

### 3.3 Prevalence of influenza vaccination according to age, sex, and smoking status in participants with diabetes

**Figure 3:**
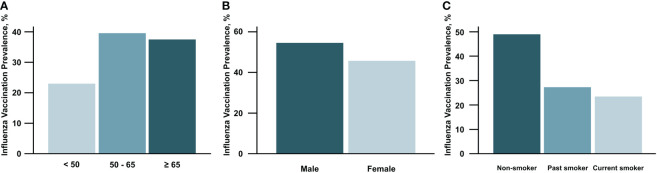
Prevalence of influenza vaccination according to age **(A)**, sex **(B)**, and smoking status **(C)** in participants with diabetes mellitus.

Comparison of prevalence of influenza vaccination according to clinical characteristics in patients with diabetes is shown in **Figure 3**. Subjects with age between 50 and 65 showed the highest prevalence, and those under 50 noted the lowest prevalence of vaccination. Male patients were a higher rate of vaccination than females. Regarding smoking status, the vaccination rate tended to decrease in the order of non-smoker, past smoker, and current smoker.

### 3.4 Prevalence of influenza vaccination according to glycemic control in participants with diabetes

**Figure 4:**
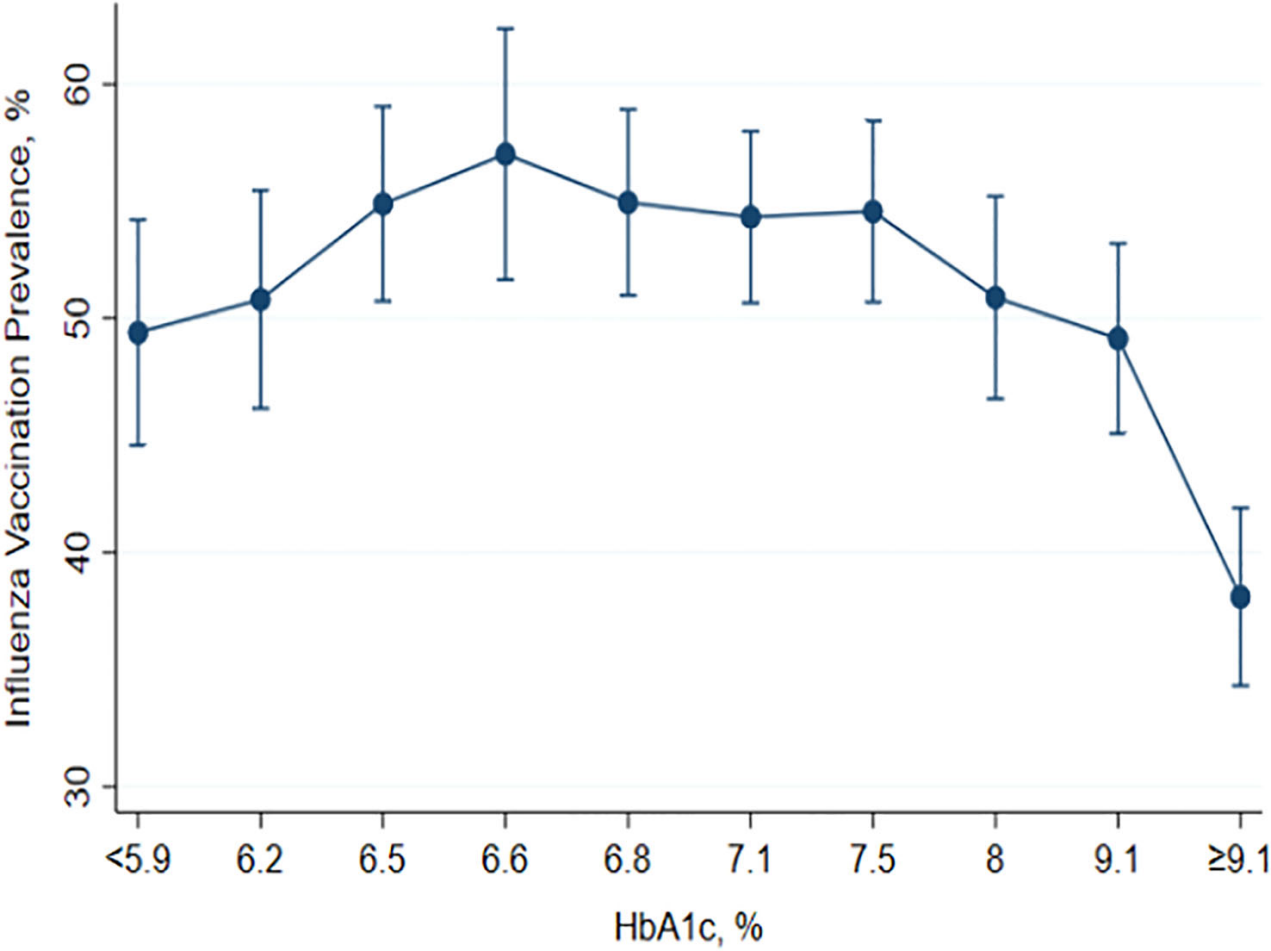
Prevalence of influenza vaccination according to glycemic control in participants with diabetes mellitus.

**Figure 4** summarizes the prevalence of influenza vaccination according to glycemic control in patients with diabetes. Relatively higher prevalence was observed in patients with HbA1c between 6.0% and 7.5%. The patients with HbA1c ≥ 9% showed the lowest prevalence of influenza vaccination (P < 0.001).

### 3.5 Factors associated with influenza unvaccinated status

To evaluate the factors associated with influenza vaccination status, a multivariable logistic regression analysis was performed (**Table 2**). Younger age was found to be associated with influenza unvaccinated status (adjusted hazard ratio (aHR) [95% CI] for age 11.29 [8.63–14.75] for < 50 years; 6.16 [5.21–7.29] for 50–65 years). Male (aHR 1.67 [1.52–1.87]), current smoker (aHR 1.31 [1.00–1.72]), and lower-income status (aHR 1.46 [1.17–1.84]) showed a significant association with unvaccinated status. A poorer glycemic control with HbA1c ≥ 9% was found to be correlated with unvaccinated status (aHR 1.48 [1.15–1.90]). In contrast, a higher education level (aHR 1.30 [1.01–1.67] for college graduate or higher**)** and comorbidities such as hypertension (aHR 0.84 [0.72–0.98]), cardiovascular disease (aHR 0.64 [0.48–0.85]), and chronic kidney disease (aHR 0.66 [0.51– 0.85]) showed a negative association with unvaccinated status.

**Table 2:** Factors associated with influenza unvaccinated status in patients with diabetes mellitus.

	Univariable		Multivariable		
	OR (95% CI)	P value	OR (95% CI)	P value	
Age, years					
< 50	14.58 (12.28, 17.30)	<0.001	11.29 (8.63, 14.75)	<0.001	
50 – 65	6.95 (6.11, 7.91)	<0.001	6.16 (5.21, 7.29)	<0.001	
≥ 65	Reference		Reference		
Sex, male	1.50 (1.46, 1.56)	<0.001	1.67 (1.52, 1.87)	0.002	
BMI, kg/m^2^					
Underweight (< 18.5)	1.18 (0.77, 1.81)	0.448	1.19 (0.60, 2.36)	0.621	
Normal weight (18.5–24.9)	Reference		Reference		
Overweight/obesity (≥25)	1.16 (1.05, 1.28)	0.004	0.96 (0.82, 1.11)	0.557	
Smoking status					
Non-smoker	Reference		Reference		
Past smoker	1.34 (1.19, 1.52)	<0.001	1.01 (0.78, 1.30)	0.939	
Current smoker	2.62 (2.29, 3.00)	<0.001	1.31 (1.00, 1.72)	0.047	
Marital status					
Unmarried	Reference		Reference		
Married	0.32 (0.24, 0.44)	<0.001	0.80 (0.48, 1.33)	0.395	
Widowed/Separated/Divorced	0.13 (0.10, 0.18)	<0.001	0.70 (0.41, 1.20)	0.192	
Income					
Low	1.46 (1.25, 1.69)	<0.001	1.46 (1.17, 1.84)	0.001	
Intermediate	1.19 (1.03, 1.36)	0.015	1.18 (0.97, 1.44)	0.105	
High	Reference		Reference		
Education					
Elementary school graduate	Reference		Reference		
Middle/High school graduate	2.64 (2.35, 2.97)	<0.001	1.09 (0.91, 1.30)	0.350	
College graduate or higher	3.58 (3.07, 4.19)	<0.001	1.30 (1.01, 1.67)	0.042	
Regular walking	1.18 (1.04, 1.34)	0.010	0.75 (0.63, 0.90)	0.002	
Any respiratory symptoms	0.86 (0.69, 1.07)	0.181	0.79 (0.59, 1.05)	0.102	
Physical limitation	0.52 (0.46, 0.60)	<0.001	0.81 (0.64, 1.02)	0.075	
EQ-5D	13.30 (8.77, 20.18)	<0.001	0.94 (0.50, 1.80)	0.861	
HbA1c					
< 9%	Reference		Reference		
≥ 9%	1.81 (1.53, 2.14)	<0.001	1.48 (1.15, 1.90)	0.002	
Comorbidities					
Pulmonary TB	0.78 (0.61, 0.99)	0.038	0.87 (0.63, 1.21)	0.412	
Hypertension	0.50 (0.45, 0.55)	<0.001	0.84 (0.72, 0.98)	0.028	
Dyslipidemia	1.06 (0.93, 1.19)	0.392	1.08 (0.89, 1.30)	0.436	
Cardiovascular disease^*^	0.45 (0.36, 0.57)	<0.001	0.64 (0.48, 0.85)	0.002	
Stroke	0.49 (0.39, 0.62)	<0.001	0.92 (0.66, 1.30)	0.640	
Chronic kidney disease	0.34 (0.28, 0.40)	<0.001	0.66 (0.51, 0.85)	0.001	

Data are presented as odds ratio (95% confidence interval).

^*^Cardiovascular disease was defined as myocardial infarction or angina.

BMI, body mass index; TB, tuberculosis.

## 4 Discussion

As a result of analyzing the prevalence of influenza vaccination for 13 years in participants with diabetes using data from Korea NHANES, the vaccination rate showed a tendency to increase every year, reaching almost 60% in 2019, which was higher than the rate in participants without diabetes. However, vaccination rates were significantly lower in certain groups of younger age, male sex, current smokers, low income-level, and high education level. Notably, poorer glycemic control (HbA1c ≥ 9%) showed a significant association with unvaccinated status.

It is well-known that diabetes is a risk factor for higher susceptibility to bacterial, viral, or fungal infection (22). Furthermore, patients with diabetes showed poor infection outcomes such as hospitalization and mortality (23, 24). Influenza is one of the most common respiratory viruses resulting in annual epidemics. Influenza infection causes substantial mortality in some subjects, especially in elderly or chronic diseases such as diabetes (6). Previous studies have shown that patients with diabetes have higher infection-related morbidity and mortality during influenza infection than those without diabetes (22, 25). In another study, a higher risk of admission to the intensive care unit was observed in patients with diabetes during the influenza pandemic (10). Therefore, several guidelines including American Diabetic Association, the European Association for the Study of Diabetes, and Korean Diabetes Association recommend that patients with diabetes ≥ 6 months of age who do not have a contraindication should receive the influenza vaccination annually (13, 26, 27).

As shown in the previous studies, our study showed that influenza vaccination rate is higher in subjects with diabetes compared with those without diabetes (28). Extending the previous findings, our study provided a long-term trend of vaccination rates in patients with diabetes. Although the prevalence of vaccination rate steadily increased during the observation period, the most recent rate of vaccination (2019) in patients with diabetes was 59.8%, suggesting there is still unmet need to increase influenza vaccination rate in this population. The World Health Organization (WHO) recommends a vaccination coverage rate of at least 75% in the vulnerable population for effective prevention against influenza (29). From this view, the clinical relevance of our study is that we revealed various factors associated with unvaccinated status, which might be helpful for physicians and health policy managers to make some enhanced strategies to increase vaccination rate in subjects with diabetes.

Our study showed younger age, male sex, current smoker, low-income status, and high education level showed significant factors affecting vaccination. Some previous studies evaluated the association between clinical characteristics and influenza vaccination coverage in patients with diabetes. In a study performed in the United States, a low vaccination rate was reported in some vulnerable sociodemographic groups such as younger adults (18−64 years), non-Hispanic Black adults, individuals without insurance, those from low-income families, and individuals lacking access to usual care (30). Another study conducted in Thailand also showed similar results: a different vaccination rate was shown according to age, sex, and comorbidities (31). Other studies in Korea reported that age and economic activity were associated with influenza vaccination (2, 28). Our findings are consistent with recent studies that decrease in vaccine coverage rates are related to young age and lower-income levels. Besides these factors, our study showed that males and current smokers are important factors associated with unvaccinated status. Considering that these two factors are significant predictors of poor outcomes of influenza (32, 33), substantial efforts are needed to increase influenza vaccination rate in these groups. Considering that education levels are generally correlated with income levels, it is very interesting that high education level was associated with unvaccinated status in patients with diabetes. Previous studies have consistently demonstrated similar results (34–36). The results from a recent study conducted in Korea also showed that a lower education level was associated with higher influenza vaccination coverage (37). It might be that people with low education levels have the need for preventive management because they have more possibility to work in fields that require more physical activities and less social support. This may partly explain the reasons for the results seen in our study. Since the reasons why high education levels are associated with low vaccination rates are still unclear, future studies are needed to improve the vaccination rate in this subpopulation.

For the first time to the best of our knowledge, it is noteworthy that our study analyzed the prevalence of influenza vaccination according to glycemic control in patients with diabetes. Although not fully elucidated, several factors have been suggested to explain the underlying mechanisms of increased risk of infection in patients with diabetes. Chronic inflammatory conditions associated with obesity, hyperlipidemia, and insulin resistance in diabetes are thought to be relevant (38, 39). Moreover, it is well-known that poor glycemic control was associated with an increased risk of infection-related morbidity and mortality in patients with diabetes (40, 41). Thus, influenza vaccination would be more important in patients with poor glycemic control. However, in our study, the patients with poor glycemic control, referred to as higher HbA1c, showed lower vaccination rates. Since our study is observational, the reason for this phenomenon is not fully explainable. We carefully suggest that low compliance to medical advice, often found in patients with poor glycemic control, might be an explanation for the lower vaccination rate in this population.

During the COVID-19 pandemic, a pattern of decreased influenza transmission has been observed worldwide. However, as social distancing relaxes and people do not wear face masks, the transmission of both influenza and COVID-19 can be expected to increase (42). Accordingly, increasing influenza and COVID-19 vaccinations carry heightened importance to minimize the tremendously harmful effects of these viruses on patients with diabetes.

One of the strengths of this study is the inclusion of a large population in the analysis because of the use of Korea NHANES data, which were taken from a nationwide survey sample showing a high response rate. In addition, the investigation was focused on glycemic control, which is an important factor in patients with diabetes. However, there are some limitations to this study. First, we did not classify type of diabetes due to the absence of information in Korea NHNES. Since Korea NHANES is a representative study designed to select samples that can represent the overall Korean population, both type 1 and type 2 diabetes subjects are thought to be enrolled in this study. However, since the prevalence of type 1 diabetes is very low (< 0.1%) in Korea (43), most of our study subjects are thought to have type 2 diabetes. Second, the potential recall bias might have affected our results because of self-reported influenza vaccination information in this database, which may underestimate or overestimate the true prevalence of influenza vaccination. Third, it is challenging to validate the causality due to cross-sectional data. Fourth, the influenza vaccination policies may differ by country. For example, in Korea, free influenza vaccination has been provided for all subjects aged 65 or older since 2005. Thus, our study results might not be applicable to other countries with different influenza vaccination policies.

In conclusion, the influenza vaccination rate is low in subjects with diabetes who are younger, males, have low-income levels, have high education levels, and those with poor glycemic control. Considering the risk-benefits of influenza vaccination in patients with diabetes, physicians should recognize the importance of immunization in diabetes and effort to increase vaccination rates in patients with diabetes, especially in low vaccination rate groups.

## Data availability statement

The raw data supporting the conclusions of this article will be made available by the authors, without undue reservation.

## Ethics statement

The studies involving human participants were reviewed and approved by The study protocol was approved by the Institutional Review Board of Chungbuk National University Hospital (application no.2022-03-003). The ethics committee waived the requirement of written informed consent for participation

## Author contributions

Conception or design: HL and SP. Acquisition, analysis, or interpretation of data: BY and SG. Drafting the work or revising: D-HL, BY, SP, and HL. Final approval of the manuscript: D-HL, BY, SG, E-GK, YK, HK, YC, HJ, and SP and HL. All authors contributed to the article and approved the submitted version.
